# History-taking revisited: Simple techniques to foster patient collaboration, improve data attainment, and establish trust with the patient

**DOI:** 10.3205/zma001505

**Published:** 2021-09-15

**Authors:** Moshe Y. Flugelman

**Affiliations:** 1 Lady Davis Carmel Medical Center, Department of Cardiovascular Medicine, Haifa, Israel; 2 Technion, Israel Institute of Technology, Rappaport Faculty of Medicine, Haifa, Israel

**Keywords:** history taking, trust, patient centered, empathy

## Abstract

The relevance and importance of the medical interview has been challenged with improved imaging technologies, web-based medicine, and use of artificial intelligence. The medical interview has three goals:

1. Acquiring accurate medical data about the patient and the etiology of symptoms and signs,
2. learning about the patient’s personality, culture, and beliefs, and
3. creating and building trust with the patient.

Reduced human resources in the medical system and increased crowding in the interview setting, such as the emergency room and outpatient clinics, have strengthened the need for high quality and efficient interviews that fulfils the three goals of the interview.

This manuscript proposes a structured six methods that contribute to the quality and efficiency of the medical interview with special focus on learning about the patients’ life and creating trust with him.

## Zusammenfassung

Die Bedeutung und der Stellenwert des Anamnesegesprächs sind durch bessere Bildgebungstechnologien, die webbasierte Medizin und die Nutzung künstlicher Intelligenz in den Hintergrund getreten. Das Anamnesegespräch verfolgt drei Ziele:

1. Präzise medizinische Informationen über den Patienten und die Ätiologie der Symptome und Anzeichen erheben;
2. Persönlichkeit, Kultur und Überzeugungen des Patienten kennenlernen und
3. eine Vertrauensbasis mit dem Patienten schaffen und ausbauen.

Reduzierte Personalressourcen im Gesundheitssystem und größere Engstände in der Gesprächssituation, beispielsweise in der Notaufnahme und in ambulanten Kliniken, haben den Bedarf nach qualitativ hochwertigen und effizienten Gesprächen verstärkt, mit welchen sich die drei Ziele des Anamnesegesprächs erreichen lassen.

Dieses Manuskript stellt eine Struktur aus sechs methodischen Schritten vor, die zur Erhöhung der Qualität und Effizienz des Anamnesegesprächs beitragen, wobei ein besonderer Schwerpunkt darauf liegt, Informationen über das Leben des Patienten zu gewinnen und Vertrauen zu schaffen.

## Introduction

I was introduced to the art of history-taking in the book *“Interviewing the Patient”* by Engel and Morgan [1]. Implementing the concepts and techniques detailed in the book has served me well for more than 40 years of practice. This book also guides me when I teach history-taking both in the classroom and at the bedside. While basic ethical and methodological methods of medical practice have not changed significantly over the past decades, the patient-physician relationship has changed significantly with less distance and formality between patients and physicians, and a lowering of the authoritative position of the physician. These changes are due to significantly improved diagnostics and therapeutics on one hand, and the readily availability of information and social changes on the other hand all leading to patient-physician shared decisions on the diagnostic process and treatments [2], [3]. 

The importance of history-taking may seem to be reduced with improved technical diagnostics and therapeutics, but everyday practice confirms that history taking is still the most important part of acquiring accurate medical data, knowing the patient, and establishing trust. Trust is the cornerstone of patient-physician relationship and makes the patient-physician encounter more effective. Trust also makes the patients journey through illness easier with less anxiety [4], [5], [6], [7]. 

By no means the techniques outlined below can replace structured, comprehensive interview but rather provide the means for an interview that will accomplish all the goals of the interview with emphasis on knowing the patient and creating trust. Knowing the patients and creating trust are essential for getting an accurate medical history and allowing creation of a working hypothesis or obtaining the needed information for differential diagnosis of the patient’s symptoms and signs. The examples in this manuscript and the suggested methods developed during my work in Israel. It definitely reflects cultural and social features of modern Israeli society. I continuously discussed history-taking with my colleagues and students and see this skill as a life-long learning process. At the same time humanistic values and the need to know our patients and create trust with them are universal and therefore the principles of the suggested methods should resonant in medical interviews everywhere. As stated above, the focus of the current manuscript is on learning about the patient’s personality, culture, and beliefs, and creating and building trust with the patient which enables accurate medical data acquisition. 

## Methods for history taking

### 1. The 4 questions

There are several characteristics that define each of us as individuals. The perspective of philosophical and humanistic disciplines may suggest avoidance of definitions. However, from a pragmatic point of view, we are technically as individuals differentiated from others based on the followings: name, address of residence, family status, and occupation. While other characteristics also define us as individuals such as hobbies and personal believes, the former four are universal and usually are not potentially associated with issues that may produce inconvenience to the patient and to the physician. After asking these four questions using the below detailed techniques, we should have a good sense of the patient in his natural habitat. Importantly, even if we make errors with generalizations and classifications, we can modify our conclusions throughout the interview if additional data do not fit into our initial assessment of the patient. 

Only if we have a genuine interest in the patient can we create a valid understanding of the patient’s habitat. When we identify a relevant or interesting point (such as an unusual name), we should follow the initial question with supplemental questions such as what the origin of the name is (see the chain reaction below). Showing keen interest in the patient’s individuality delivers a clear message: I care about you and I want to get to know you. The timing for asking the four question depends on the way the interview goes. As always, the first question the patient should be addressed with is “How are you?”. Also, if you know the patients name there is no need to ask him the name but rather use the name as a trigger to a sequence of questions.

#### 2. The chain reaction

The chain reaction method refers to a conscious use of a line of questions focusing on a single issue using the answer to formulate a question that adds additional information with higher resolution. A simple example is the following question after hearing the family name of the patient. The follow-up question is what is the origin of the name? The next question is when did your family move to the current location? And then the following question is: do you keep the traditions of the original location or do you support the sport teams or other topics related to the original location of your family. At the end of sequence of 4 questions, which are a part of a “natural” conversation (not a questionnaire), the physician has learnt a significant amount of information about the patient, while the patient feels that the physician is interested in him/her and is able to create a seemingly casual conversion with him/her. This is extremely important as will be detailed in method number 4.

#### 3. Identification of standard deviations

Physicians are taught to listen, observe, and identify pathologies. The greatest challenge is to identify what is normal and what is deviating from normal, and therefore is potentially pathological. While the boundaries of normal and pathological in physical examination, laboratory tests, and imaging are well defined, the normal and the deviation from the normal in patients’ personality, cultural habits, and beliefs are less well defined. Personality, culture, and beliefs are extremely important in creating trust between the patient and the physician. The structured interview allows us to learn about the patient’s life, and tailor the diagnostic and therapeutic processes to the patient. The four questions above with the chain reaction technique help explore the patient as an individual. A physician with a large cultural vocabulary can identify what is normal for the specific patient and what is a standard deviation that can be defined as pathological. For example, an 82-year-old patient describes that he has four offspring. The following question is where does your offspring reside? The answer is that three live out-of-town and one lives with me. The age of the offspring that lives with him is 35 years old. This is a standard deviation in most western countries. Additional questions reveal that the 35 years old son is mentally handicapped, and the patient’s greatest concern is ongoing care of this son while he is in the hospital as his son cannot function on his own. This fact provides a new perspective into the patient’s medical situation and dictates a different approach than the approach that would be adopted if the son could support the father during recovery. Shorter hospitalization, help with the son or delaying intervention till an arrangement for the son is possible should be considered. Attention to similar deviations in the four above questions, and use of chain reaction will provide us with an in-depth understanding of the patient’s concerns and psychology. 

#### 4. Equality standing

The conventional relationship between a physician and a patient is of a person-in-need and a person that provides help. Such relationship creates inequality between the patient and the physician. In addition, patients are often assumed to be socially vulnerable and weak in contrast to the strong and authoritative physician. This inequality is echoed by repeated requests for physicians to speak at eye level and a general public distrust of figures of authority. To overcome this inequality and improve the trust and relationship with a patient, the following technique can be employed: when interviewing the patient, the physician should show that he/she shares interests with the patient. Sharing can be accomplished by expressing interest in or knowledge of the patient’s profession. For example, if the patient is a garbage collector, ask him/her about the safety of riding in garbage trucks or whether he/she has found valuables in the garbage. Another strategy is to share common interests or acquaintances with the patient. In many situations, a common interest is sports, music, culture or food. An acquaintance can be a famous person from the patient’s neighborhood or someone from work. Sharing an interest such as a sports team or talking about someone famous from the neighborhood of the patient, or an event that is related to the patient´s neighborhood or working place, can create an informal favorable atmosphere during the encounter. An example can be the garbage collector. After asking him/her about the safety of the work and finding valuables, the physician can ask about a famous football player that comes from the same neighborhood. The patient remotely knew this player from childhood and feels proud that he/she can contribute information about the player that the physician did not know. Such episodes, initiated by the physician, reduce levels of inequality and promote collaboration and trust and are other examples of use of narrative medicine tools to improve trust and promote quality of medicine [8], [9], [10]. 

#### 5. What ends well is all well

The answers to chain reaction sets sequel of questions may often contribute to the inherent inequality in the patient-physician relationship. The patient responses to the four initial questions may be that he is not married, unemployed and soon homeless. From the patient’s point-of-view he is inferior to the educated, socially appreciated and high-earning physician. In order to minimize inequality every sequel of questions must end with an answer that portrays the patient in a socially favorable light.

The sequel of the four questions ends with negative feelings for the patient. The physician then asks him whether he has friends in town, and the answer is no friends in town, a continuation of unfavorable sequelae. The next question is about relatives, and the answer is yes, I do have a cousin in the neighboring city. A short comment by the physician that relates to the advantages of having a relative not so far away can terminate the negative sequel and “what ends well is all well”, the initiation of the next sequel is with a positive sentiment. 

#### 6. Be gentle, be aware 

During the interview the physician should be sensitive to social and cultural perceptions employed by the patient. As an experienced physician tends to categorize patients into patterns and employ pattern recognition as a diagnostic tool, the physician is taking risks of making errors [11], [12]. Nevertheless, immediately after greeting the patient, physicians should make assumptions and base their interactions upon their assumption and modify their behaviors based on these assumptions. Of course, physicians are aware that they make errors, and with new information the assumptions should be modified. For example, in some societies having no children is a significant disadvantage and may symbolize a failure while in other societies, having or not having children is a respectable choice. Therefore, when interviewing a person who may be sensitive to the issue of having children, this issue must be explored gently and not with a direct question. For example, a person who is part of a community in which it is customary to have large family should be addressed with the question “does he have a family” and not by a direct question “how many children do you have”. Obviously if the patient is surrounded by children when you see him first, the question can be direct, but otherwise this may be a distressing issue that may increase inequality and resentment. 

## Discussion

The science and practice of medicine have changed enormously over the past decades. Improved technologies and therapies have completely altered the diagnostic process and the outcome of most diseases. Availability of information and socio-economic changes have also added to the dynamic transformation of the encounter between patients and physicians. Previously an intimate, long encounter, often dealing with non-medical issues and with authoritative decisions made by the physician has nowadays been transformed into an encounter which is time limited, with decisions taken by physician and patients together [2], [3]. The immediate outcome of this transformation is the devaluation of the centrality and importance of the medical interview [13]. Diagnosis of many diseases depends on history alone; angina pectoris, chronic bronchitis, depression as just a few examples. Yet, many physicians and patients alike seek laboratory and imaging tests to establish the diagnosis instead of relying on a quality interview. Complete reliance on imaging and laboratory tests with detachment from patient’s history and physical findings can lead to significant and life-threatening errors and unjustified use of resources [14], Making decisions on treatments without through knowledge of the patient, his personality, and beliefs can lead to frustration and futile medical care [15].

Time resource in most patient encounters is limited, which is often used by medical personnel as a justification for less rigor and attention to history-taking [13]. Physicians in training and students often complain that there is insufficient time for detailed history taking, and therefore they rely on laboratory and imaging findings in the initial diagnostic process. While such approach may, or may not be effective in making diagnoses, it ignores the two other roles of history taking, namely, knowing the patient and creating trust. Physicians use multiple skills when interviewing a patient. The craftsmanship of conducting an interview should be integrated with empathy and authenticity to achieve the above detailed goals both in undergraduate and in postgraduate medical education and training. Definition of empathy is complex but it can be defined as “the capacity to

1. be affected by and share the emotional state of another,
2. assess the reasons for the other’s state, and
3. identify with the other, adopting his or her perspective” [16].

Its impact of quality of care and patient’s satisfaction is well established [17], [18], [19].

Communication via social media as the main human interaction is being criticized as the cause for alienation and lack of social skills. Personal privacy may limit the intimacy in usual social encounters but in medical encounter intimacy is a must; without intimacy it is impossible to create compassion and empathy. When judged in cultural and social measures, medical interview may sound intrusive. Asking a patient about his family, occupation, beliefs or other intimate issues, may seem insensitive and unnecessary. Medical interview is a unique situation in which a limited time is given to create bilateral intimate relationship that will allow accurate decision making and emotional exchange that will help alleviate suffering [17], [18], [19]. In very rare occasions, patients will be offended by such intimate questions and will resent intimate relationship with the physician. In such circumstances the interview should be adjusted to patient’s personality and emotional needs. It is unwise and potentially risky to make decisions about patients that we do not know and that do not trust us. The intimate relationship between the physician and the patients that are the basis for trust can be only created at bedside [16], [19]. 

The 6 methods described above were developed based on the understanding that the encounter is occurring at different layers; information layer, psychological, cultural, and social layers. The methods address several major issues that physicians often are dealing with. The most urgent one is a need to produce trust with the patient very rapidly in order to win his cooperation. The second one is to overcome cultural and socioeconomic variability and differences, to create a common language with the patient and to understand the expectations of the patient. Finally, to build a positive and healing atmosphere in which the patient and the physician act together. Some of the 6 techniques are used intuitively by experienced physicians. The structured presentation of these techniques provides a framework for teaching these techniques. 

In the manuscript the only reference is to the patient; however, the same 6 methods should be employed when speaking to the family of the patient. The methods could and should also be used when employing telemedicine methods once privacy of the encounter is secured. 

In summary, the six methods described provide useful means to achieve a quality interview, which is time-efficient, creates trust with the patient, and provides reliable understanding of the patient’s culture and personality. An empirical evaluation of the impact of the proposed methods should be addressed by educational researchers with qualitative and quantitative methods in controlled settings as well as in the clinical field.

## Acknowledgements

I thank Prof. Martin Fischer who encouraged me to write this manuscript and for his thoughtful and educational comments. I am grateful to Prof. Bradley Strauss who helped me write this manuscript and Dr. Robert Gluck that reviewed the manuscript and enriched its content. I thank Prof. Simon Marom for his thoughtful review and finally, I thank all my teachers who taught me over the years and all the patients who I took care of and who continue to teach me on how to take history. 

## Competing interests

The author declares that he has no competing interests.
